# Prevalence of Gastrointestinal Symptoms and Fecal Viral Shedding in Patients With Coronavirus Disease 2019: A Systematic Review and Meta-analysis

**DOI:** 10.1001/jamanetworkopen.2020.11335

**Published:** 2020-06-11

**Authors:** Sravanthi Parasa, Madhav Desai, Viveksandeep Thoguluva Chandrasekar, Harsh K. Patel, Kevin F. Kennedy, Thomas Roesch, Marco Spadaccini, Matteo Colombo, Roberto Gabbiadini, Everson L. A. Artifon, Alessandro Repici, Prateek Sharma

**Affiliations:** 1 Swedish Medical Center, Seattle, Washington; 2 Kansas City Veterans Affairs Medical Center, Kansas City, Missouri; 3 University of Kansas Medical Center, Kansas City; 4 Ochsner Clinic Foundation, New Orleans, Louisiana; 5 St Luke’s Medical Center, Kansas City, Missouri; 6 Department of Interdisciplinary Endoscopy, University Hospital Hamburg-Eppendorf, Hamburg, Germany; 7 Instituto Clinico Humanitas, Department of Endoscopy, Humanitas University School of Medicine, Milano, Italy; 8 Division of Gastrointestinal Endoscopy, University of São Paulo School of Medicine, São Paulo, Brazil

## Abstract

**Importance:**

Coronavirus disease 2019 (COVID-19) is a global pandemic and can involve the gastrointestinal (GI) tract, including symptoms like diarrhea and shedding of the severe acute respiratory syndrome coronavirus 2 (SARS-CoV-2) in feces.

**Objective:**

To provide a pooled estimate of GI symptoms, liver enzyme levels outside reference ranges, and fecal tests positive for SARS-CoV-2 among patients with COVID-19.

**Data Sources:**

An electronic literature search was performed for published (using MEDLINE/PubMed and Embase) and preprint (using bioRxiv and medRxiv) studies of interest conducted from November 1, 2019, to March 30, 2020. Search terms included “COVID-19,” “SARS-Cov-2,” and/or “novel coronavirus.”

**Study Selection:**

Eligible studies were those including patients with SARS-CoV-2 infection who reported GI symptoms.

**Data Extraction and Synthesis:**

Data on patients with GI symptoms (ie, diarrhea, nausea, or vomiting), liver enzyme level changes, and fecal shedding of virus were extracted. Quality of studies was examined using methodological index for nonrandomized studies. Pooled estimates (%) were reported with 95% CIs with level of heterogeneity (*I*^2^).

**Main Outcomes and Measures:**

Study and patient characteristics with pooled detection rates for diarrhea, nausea or vomiting, liver enzyme levels outside reference ranges, and SARS-CoV-2 positivity in feces tests were analyzed.

**Results:**

Of 1484 records reviewed, 23 published and 6 preprint studies were included in the analysis, with a total of 4805 patients (mean [SD] age, 52.2 [14.8] years; 1598 [33.2%] women) with COVID-19. The pooled rates were 7.4% (95% CI, 4.3%-12.2%) of patients reporting diarrhea and 4.6% (95% CI, 2.6%-8.0%) of patients reporting nausea or vomiting. The pooled rate for aspartate aminotransferase levels outside reference ranges was 20% (95% CI, 15.3%-25.6%) of patients, and the pooled rate for alanine aminotransferase levels outside reference ranges was 14.6% (95% CI, 12.8%-16.6%) of patients. Fecal tests that were positive for SARS-CoV-2 were reported in 8 studies, and viral RNA shedding was detected in feces in 40.5% (95% CI, 27.4%-55.1%) of patients. There was high level of heterogeneity (*I*^2^ = 94%), but no statistically significant publication bias noted.

**Conclusions and Relevance:**

These findings suggest that that 12% of patients with COVID-19 will manifest GI symptoms; however, SAR-CoV-2 shedding was observed in 40.5% of patients with confirmed SARS-CoV-2 infection. This highlights the need to better understand what measures are needed to prevent further spread of this highly contagious pathogen.

## Key Points

**Question:**

What are the incidence rates of gastrointestinal symptoms among patients with severe acute respiratory syndrome coronavirus 2 (SARS-CoV-2) infection?

**Findings:**

This systematic review and meta-analysis of 23 published and 6 preprint studies found that approximately 12% of patients with SARS-CoV-2 infection reported gastrointestinal symptoms, including diarrhea, nausea, and vomiting. Liver enzyme levels outside reference ranges were observed in 15% to 20% of patients, and SARS-CoV-2 RNA shedding in stool was detected in up to 41% of patients.

**Meaning:**

These findings suggest that patients with SARS-CoV-2 infection can present with gastrointestinal symptoms with possible fecal-oral route of transmission due to the presence of viral RNA in stool.

## Teaser

This systematic review and meta-analysis examines the prevalence of reported gastrointestinal symptoms in patients with coronavirus disease 2019 and of viral RNA shedding detected in these patients’ stool.

## Introduction

A global pandemic emerged in December 2019 from a novel severe acute respiratory syndrome coronavirus 2 (SARS-CoV-2).^1^ Phylogenetics of this virus indicate that SARS-CoV-2 is a single-stranded positive-sense RNA virus, has 79.5% homology with SARS-CoV, and is closely related to bat-derived SARS-like coronaviruses.^2^ A recent large case series of 72 314 infected individuals in China showed an estimated 14% with severe disease and a case-fatality rate of 2.3%.^3,4^

The coronaviruses are a common source of upper respiratory, gastrointestinal, and central nervous system infections in humans and other mammals.^5^ SARS-CoV-2 is highly homologous to SARS-CoV, and similar to SARS-CoV, angiotensin converting enzyme 2 (ACE2) is also the cellular entry receptor of SARS-CoV-2.^6,7,8^ Since ACE2 is found in the absorptive enterocytes of the ileum and colon, these absorptive enterocytes can be infected by a host of viruses, including coronavirus, rotavirus, and noroviruses, thereby resulting in diarrhea.^7,9^ A few bioinformatics studies^10,11^ found that ACE2-expressing intestinal epithelium cells might be at increased risk of attack by SARS-CoV-2 and that ACE2 was highly expressed in the small intestine, especially in proximal and distal enterocytes. Hence, the digestive system can be invaded by SARS-CoV-2 and serve as a route of infection.

In fact, the first reported patient with coronavirus disease 2019 (COVID-19) in the US reported gastrointestinal (GI) symptoms of loose bowel movements and abdominal discomfort.^12^ The patient’s stool and respiratory specimens were found to be positive for SARS-CoV-2 by real-time reverse transcription–polymerase chain reaction (RT-PCR).^12^ This raises the question of inadvertent human-to-human transmission via the fecal route despite public health emphasis on droplet transmission and precautions for contact with respiratory secretions. Hence, additional information and understanding the involvement of the digestive system in transmission of COVID-19 during this pandemic would be useful.

Our objective was to determine the prevalence of GI symptoms at presentation of COVID-19 and viral shedding in stool of patients with confirmed SARS-CoV-2 infection based on published literature. We have also included data from preprint publications as a separate category to provide a concise review of the existing knowledge as this global emergency unfolds.

## Methods

This systematic review and meta-analysis was performed according to the Preferred Reporting Items for Systematic Reviews and Meta-analyses (PRISMA) reporting guideline.^13^ Per institutional review board policy of the University of Utah, institutional review board approval was not required for this study since it did not involve any direct human participant research.

### Search Strategy

**Figure 1.**
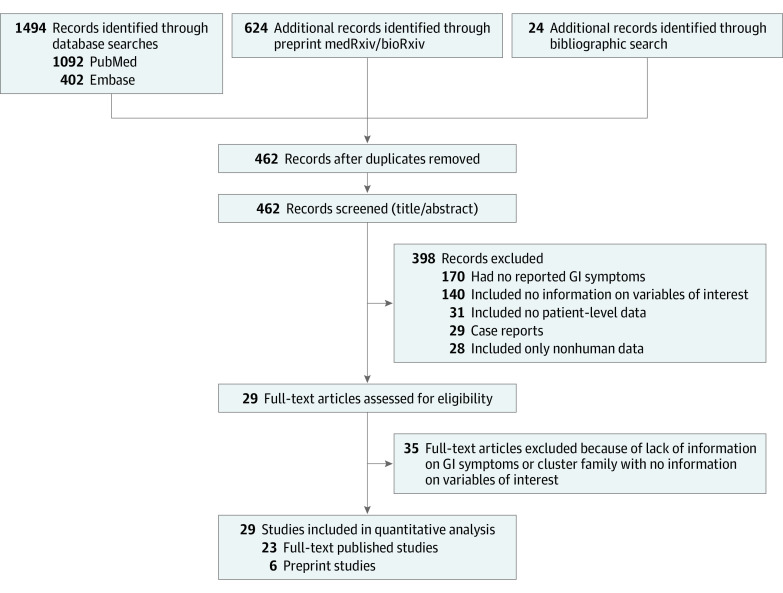
PRISMA Flow Diagram Depicting Study Selection Process GI indicates gastrointestinal.

We performed a literature search combining Medical Subject Headings (MeSH) terms and keyword searches using MEDLINE/PubMed and Embase to find studies published from November 1, 2019, to March 30, 2020, using the terms “COVID-19,” “SARS-Cov-2,” and/or “novel coronavirus.” We also queried the newly developed artificial intelligence–enabled search engine, CORD-19 (Allen Institute for Artificial Intelligence), which has partnered with leading research groups to prepare and distribute the COVID-19 Open Research Data set, a free resource of more than 29 000 scholarly articles, and also archival services, such as bioRxiv and medRxiv.^14,15^ A secondary search through references of published studies was also performed. The reference lists of all studies were reviewed for additional sources of data. Duplicate citations were removed, and all remaining studies were screened for eligibility by review of their titles and abstracts. The search strategy and method used for selection of studies is presented in Figure 1.

### Eligibility Criteria

Eligibility criteria included observational studies reporting clinical symptoms at presentation in patients with COVID-19 (determined by nasopharyngeal swabs which were positive for SARS-CoV-2 in PCR) to estimate the prevalence of GI symptoms when present and observational studies providing data regarding RNA detection or isolation of SARS-CoV-2 in stool samples of patients with COVID-19. Studies in abstract form only were excluded owing to limited available information satisfying eligibility for analysis of our outcomes of interest. After application of these criteria, an in-depth review of the remaining studies was conducted along with data extraction into an Excel spreadsheet (Microsoft). Two investigators (S.P. and M.D.) independently performed the search.

### Data Extraction and Quality Appraisal

Data were extracted and recorded from all studies as study type, author, year of publication, country and site or setting, age, sex, number of patients, primary aim of the study, timing of data collection, symptom prevalence (including cough, diarrhea, and nausea or vomiting among primary symptoms), laboratory test results (including liver enzyme levels, such as aspartate aminotransferase [AST], alanine aminotransferase [ALT], alkaline phosphatase [ALP], and total bilirubin), follow-up determination, collection of stool viral RNA load, and performance of endoscopy. Quality of included studies was assessed using a validated quality grading instrument, methodological index for non-randomized studies (MINORS).^16^ The following criteria were evaluated and a score of 0 (not present), 1 (reported but inadequate), or 2 (reported and adequate) was provided for each: clearly stated aim, inclusion of consecutive patients, prospective collection of data, end points appropriate to the aim of the study, unbiased assessment of the study end point, follow-up period appropriate to the aim of the study, loss to follow-up less than 5%, and prospective calculation of the study size.

### Statistical Analysis

The primary objective of this systematic review and meta-analysis was to assess the rates of GI symptoms (ie, diarrhea, nausea, or vomiting) reported among patients with COVID-19. This was calculated as the pooled prevalence rate of these GI symptoms individually. Studies that did not report GI symptoms were excluded from this analysis. Secondary objectives included calculating the pooled estimate rates for GI symptoms and rates of virus shedding in stool of patients with COVID-19 among published and archived studies (ie, preprint studies) as derived from COVID-19 Semantic Scholar.^12,17^ Statistical analysis was performed using R statistical software (R Project for Statistical Analysis). The extent of heterogeneity across studies was assessed using Q statistics and *I*^2^ index. For the *I*^2^ statistic, heterogeneity was defined as low (25%-50%), moderate (50%-75%), or high (>75%). Publication bias was assessed using funnel plots, and asymmetry of the funnel was evaluated with Egger regression test. *P* values were 2-sided, and *P* < .05 was considered statistically significant. Data were analyzed from December 2019 to March 2020.

## Results

### Study Characteristics

The initial search using the MeSH terms yielded 1484 studies. After screening titles, abstracts, and full texts and for the studies in preprint version derived from COVID-19 Semantic Scholar, the selection was reduced to 614 studies. After further screening and applying the inclusion and exclusion criteria, 21 publications^10,14,17,18,19,20,21,22,23,24,25,26,27,28,29,30,31,32,33,34^ and 8 preprint studies^1,3,35,36,37,38,39,40^ were included for the final analysis (2 preprint studies were accepted in the time between initial analysis and final analysis) (Table). Most of these studies were single-arm observational studies with retrospective reporting of data from hospitalized patients, and most studies were reported from mainland China. The primary method of data collection was extraction of information from electronic medical records of hospitalized patients. Most studies collected data on adults, 1 study collected data on pregnant patients,^18^ and 3 studies included data on children younger than 18 years.^19,35,36^ One study reported information on health care personnel.^17^

**Table.** Study and Patient Characteristics of Included Studies

Source	Initial publication date^a^	Site (city or province, country)	Time period	Population	Patients, No.	Age	No. (%)						
							Women	Symptoms			Elevated serum level		
								Diarrhea	Nausea or vomiting	Cough	ALT	AST	Total bilirubin
**Published articles**													
Chen et al^18^	March 7, 2020	Zhongnan Hospital, Wuhan University, Wuhan, China	January 20-31, 2020	Pregnant women with laboratory-confirmed COVID-19 pneumonia	9	Range, 26-40 y	9 (100)	1 (11)	0	4 (44)	3 (33)	3 (33)	NA
Zhang et al^10^	March 3, 2020	Jinhua Hospital of Zhejiang, China University, Jinhua, China	January 27-February 10, 2020	Patients with laboratory-confirmed COVID-19 pneumonia	14	Median (IQR), 41 (18-87) y	7 (50)	0	0	10 (71)	NA	NA	NA
Wang et al^19^	March 2, 2020	21 hospitals in 17 cities in Shaanxi, Gansu, Ningxia, Hebei, Henan, and Shandong provinces, China	January 25-February 21, 2020	Children	31	Median (IQR), 7 y, 1 mo (6 mo-17 y)	NA	3 (9)	0	14 (45)	6 (22)	6 (22)	NA
Xiao et al^20^	March 3, 2020	Sun Yat-sen University, Guangzhou, China	February 1-14, 2020	Hospitalized patients with SARS-CoV-2 detected in stool	73	Range, 10 mo-78 y	14 (19)	26 (35.6)^b^	NA	53 (72.6)	NA	NA	NA
Ling et al^21^	February 28, 2020	Shanghai Public Health Clinical Center, Shanghai, China	January 20-February 10, 2020	All patients with COVID-19 in Shanghai region	66	Median (IQR), 44.0 (34.0-62.0) y	28 (42)	NA	NA	NA	NA	NA	NA
Liu et al^25^	February 7, 2020	9 tertiary hospitals in Hubei province, China	December 30, 2019-January 24, 2020	Patients with COVID-19 admitted to the respiratory departments	137	Median (IQR), 57 (20-83) y	76 (55)	11 (8)	NA	66 (48)	0	0	0
Yang et al^14^	February 26, 2020	Multicenter study in Wenzhou, China	January 17-February 10, 2020	Patients with SARS-CoV-2 infection confirmed via RT-PCR	149	Mean (SD), 45.1 (13.4) y	68 (46)	11 (7)	2 (1)	87 (58)	18 (12)	27 (18)	4 (3)
Xu et al^17^	February 19, 2020	7 hospitals in Zhejiang province, China	January 10-26, 2020	Hospitalized patients with laboratory-confirmed SARS-Cov-2 infection	62	Median (IQR), 41 (32-52) y	27 (44)	3 (8)	NA	50 (81)	Not reported	10 (16)	NA
Liu et al^26^	March 12, 2020	Jianghan University Hospital, Wuhan, China	January 3-11, 2020	Physicians and nurses	30	Mean (SD), 35 (8) y; range, 21-59 y	20 (66)	9 (30)	9 (30)	25 (83)	7 (23)	7 (23)	NA
Guan et al^22^	February 28, 2020	Wuhan, China	December 11, 2019-January 29, 2020	Adults with SARS-CoV-2 infection	1099	Median (IQR), 47 (35-58) y	41.9	41 (3.7)	55 (5)	744 (68)	158 (21)^c^	168 (22)^d^	76 (10)^e^
Huang et al^27^	January 24, 2020	Wuhan, China	December 16, 2019-January 2, 2020	Adult patients	41	Median (IQR), 49 (41-58) y	11 (27)	1 (3)	0	31 (76)	NA	15 (37)	NA
Chen et al^24^	January 30, 2020	Wuhan, China	January 1-20, 2020	Adult patients	99	Mean (SD). 55.5 (13.1) y	32 (32)	2 (2)	1 (1)	81 (82)	NA	NA	NA
Wang et al^23^	February 7, 2020	Zhongnan, Wuhan	January 1-28, 2020	Adult patients	138	Median (IQR), 56 (42-68) y	63 (45.7)	14 (10)	19 (14)	82 (59)	0	0	NA
Zhang et al^28^	March 12, 2020	Fever clinic in Beijing, China	January 18-February 3, 2020	All patients	9	Median (IQR), 36 (15-49) y	4 (44)	1 (11)	NA	5 (56)	NA	NA	NA
Chen et al^29^	March 12, 2020	Huazhong University of Science and Technology, Wuhan, China	January 2020	Patient admitted to the isolation ward	29	Mean, 56 y	8 (28)	4 (14)	NA	21 (72)	5 (17)	7 (24)	1 (3)
Wang et al^27^	March 16, 2020	Shanghai Public Health Clinical Center, Shanghai, China	January 21-24, 2020	Patients receiving combined Chinese and Western medicine	4	NA	1 (25)	0^f^	NA	3 (75)	NA	NA	NA
Chang et al^30^	February 2, 2020	3 hospitals in Beijing, China	January 16-29, 2020	Hospitalized patients	13	Median (IQR), 34 (34-48) y	3 (13)	1 (8)	NA	6 (46)	NA	NA	NA
Pan et al^31^	March 18, 2020	Multicenter study, Wuhan, China	January 18-February 28, 2020	Adult patients	204	Mean (SD), 54.9 (15.4) y	97 (48)	29 (14)	8 (1)	NA	0	0	0
Luo et al^32^	March 20, 2020	Wuhan, China	January 1-February 20, 2020	Hospitalized patientts	1141	Mean, 53.8 y	81 (44)	68 (6)	134 (12)	NA	NA	NA	NA
Zhou et al^33^	March 12, 2020	Wuhan, China	December 20, 2019-February 9, 2020	Medical staff and nonstaff patients	254	Median (IQR), 51 (15-87) y	139 (55)	46 (18)	21 (8)	98 (39)	NA	NA	NA
Jin et al^34^	March 24, 2020	Multicenter study, Zhijang province, China	January 17-February 8, 2020	Adult patients	651	Mean (SD), 45.6 (14.2) y	320 (49)	53 (8)	21 (3)	435 (67)	NA	NA	NA
**Preprint studies**													
Zhang et al^35^	March 16, 2020	Hubei University of Medicine, Shiyan, China	January 1-February 23, 2020	Patients aged 0-14 y	34	Median (IQR), 33 (10-94.3) mo	20 (59)	4 (12)	4 (12)	20 (59)	NA	NA	NA
Qian et al^37^^g^	February 1, 2020	Wuhan, China	January 20-February 11, 2020	NA	91	Median (IQR), 50 (36.5-57) mo	54 (59)	21 (23)	17 (19)	55 (60)	7 (8)	9 (10)	NA
Fan et al^1^^g^	February 28, 2020	Shanghai, China	January 20-31, 2020	Patients aged 15-88 y	148	Median (IQR), 50.5 (36-64) y	72 (49)	6 (4)	3 (2)	67 (45)	75 (51)	75 (51)	NA
Zhao et al^38^	March 6, 2020	First affiliated hospital of University of Science and Technology of China	January 21-February 16, 2020	Patients aged 16-91 y	75	Median (IQR), 47 (34-55) y	33 (44)	9 (9)	0	62 (83)	15 (20)	14 (19)	NA
Han et al^39^^g^	March 30, 2020	Wuhan, China	February 13-29, 2020	Hospitalized patients aged 27-92 y	206	Median (IQR), 63 (27-92) y	115 (56)	67 (33)	24 (12)	53 (26)	NA	NA	NA
Xu et al^36^^g^	March 13, 2020	Guangzhou Women and Children’s Medical Center, Guangzhou, China	January 22-February 20, 2020	Pediatric patients	10	Range, 2 mo-15 y	4 (40)	2	NA	NA	NA	NA	NA
Wu and McGoogan^3^^g^	February 24, 2020	Multicenter CDC database	December 9, 2019-February 11, 2020	72 314 case records	44 672 confirmed cases	NA	NA	NA	NA	NA	NA	NA	NA
Liu et al^40^	March 13, 2020	Union Hospital, Wuhan, China	January 16-February 15, 2020	Patients aged 23-63 y	64	Median (IQR), 35 (29-43) y	41 (64)	3 (5)	0	30 (47)	8 (13)	6 (9)	NA

Abbreviations: ALT, alanine aminotransferase; AST, aspartate aminotransferase; CDC, Chinese Center for Disease Control and Prevention; COVID-19, coronavirus disease 2019; IQR, interquartile range; NA, not available.

^a^ Some articles were presented online ahead of publication, so dates here may not match official publication dates.

^b^ An additional 10 (13.6%) experienced gastrointestinal bleeding.

^c^ Includes data for 741 patients.

^d^ Includes data for 757 patients.

^e^ Includes data for 722 patients.

^f^ Two patients (50%) reported constipation.

^g^ The article has been published since our analysis.

Overall, the studies included 4805 patients (mean [SD] age, 52.2 [14.8] years; 1598 [33.2%] women). All studies were conducted between November 1, 2019, and March 30, 2020. Eight studies provided information on viral shedding in stool.^10,17,20,21,41,42,43,44^

### Quality of Studies

Overall, most of the incorporated studies scored between 8 and 10 on the MINORS quality assessment (ie, moderate quality), with important factors being stated aims of the study, inclusion of eligible patients, and appropriately stated end points. Uniformly, sample size calculation or estimation and loss of data contributing to any attrition bias were not reported by the studies. The results of quality assessment are shown in eTable 1 in the Supplement.

### Analysis of Primary Outcomes

Of 29 publications and preprint studies included in the final analysis, 26 studies reported the occurrence of diarrhea, whereas 12 studies measured the occurrence of GI manifestations as vomiting or nausea.

**Figure 2.**
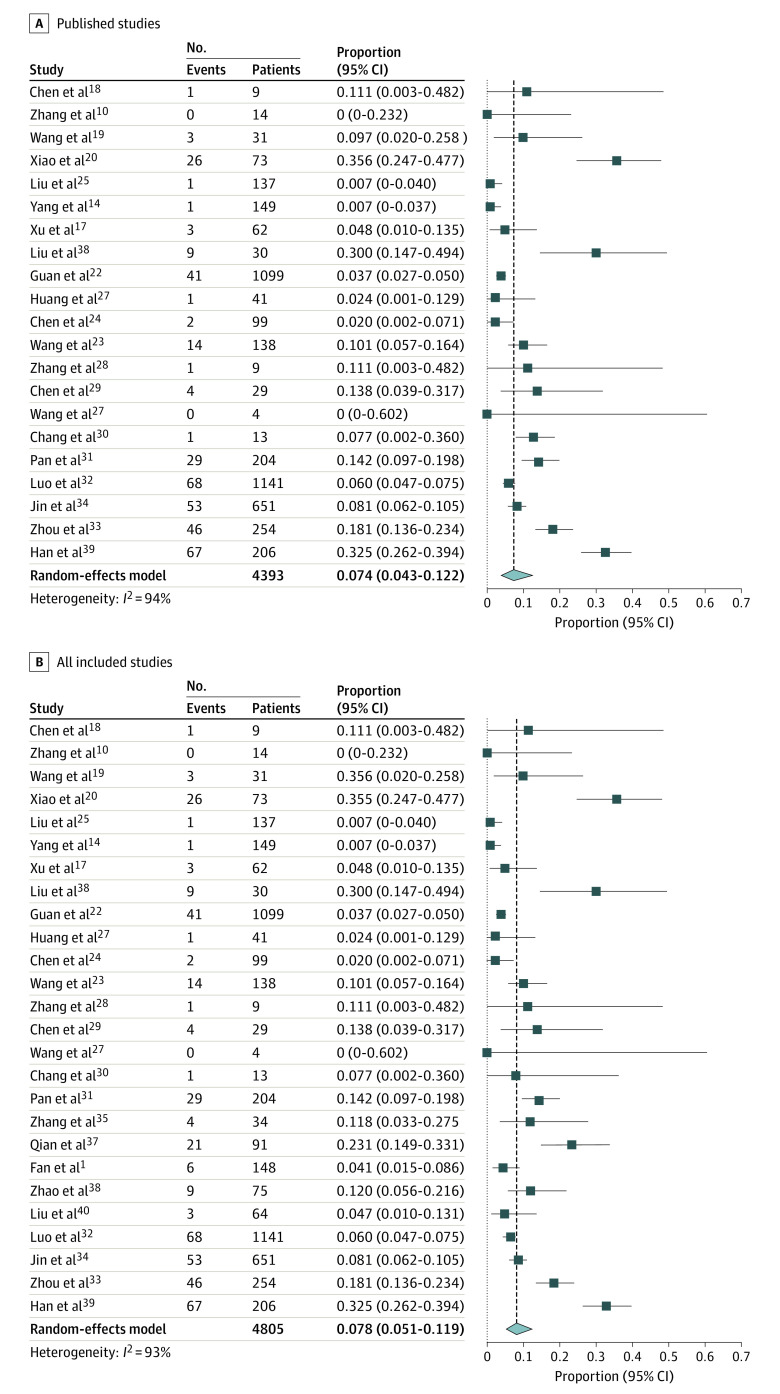
Forest Plots of Included Studies Showing Pooled Estimate of Diarrhea All studies were published or appeared as preprints in 2020. Squares indicate proportions; whiskers, 95% CI; diamond, random-effects model estimate.

The most commonly reported GI symptom at presentation was diarrhea, with pooled estimate among published studies of 7.4% (95% CI, 4.3%-12.2%) of patients (Figure 2), which includes 371 patients with diarrhea of 4393 patients from 21 studies (*I*^2^ = 94%; Egger test for bias, *P* = .64). Pooled prevalence of diarrhea when including all studies published and preprint was 7.8% (95% CI, 5.1%-11.9%%) of patients, which includes 414 patients with diarrhea of 4805 patients from 26 studies (*I*^2^ = 93% Egger test for bias, *P* = .49).

**Figure 3.**
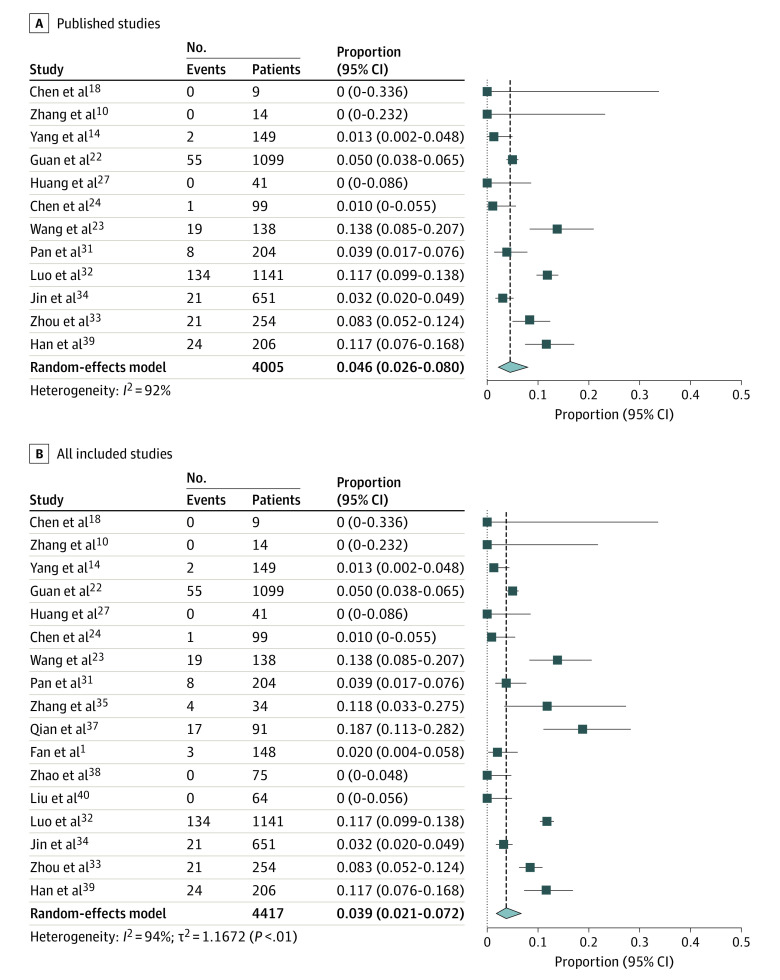
Forest Plots of Included Studies Showing Pooled Estimate of Nausea or Vomiting Symptoms All studies were published or appeared as preprints in 2020. Squares indicate proportions; whiskers, 95% CI; diamond, random-effects model estimate.

The pooled prevalence of nausea or vomiting in published studies was 4.6% (95% CI, 2.6% - 8%) of patients, including 285 patients with nausea or vomiting of 4005 patients from 12 studies (*I*^2^ = 92%; Egger test for bias, *P* = .10) (Figure 3). Pooled prevalence of nausea of vomiting was 3.9% (95% CI, 2.1%-7.2%) of patients when all studies were considered, including 309 patients with nausea or vomiting of 4417 patients from 17 studies (*I*^2^ = 94%; Egger test for bias, *P* = .25).

### Occurrence of SARS-CoV-2 Shedding in Stool

**Figure 4.**
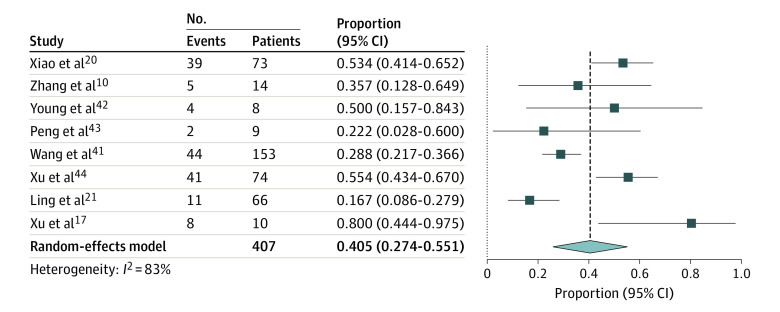
Forest Plot of Included Studies Showing Pooled Estimate of Viral Shedding in Feces All studies were published or appeared as preprints in 2020. Squares indicate proportions; whiskers, 95% CI; diamond, random-effects model estimate.

There were 8 studies that reported detection of viral RNA of SARS-CoV-2 in stool. Pooled detection of patients with fecal samples positive for SARS-CoV-2 RNA for patients who were confirmed by nasopharyngeal swab testing or respiratory secretion analysis for PCR to have COVID-19 was 40.5% (95% CI:27.4%-55.1%) of patients (Figure 4),^10,17,20,21,41,42,43,44^ including 154 patients with fecal samples positive for SARS-CoV-2 of 407 patients from 8 studies (*I*^2^ = 83%; Egger test for bias, *P* = .86). Characteristics of these studies are presented in eTable 2 in the Supplement.

In the study by Wang et al,^41^ among a total of 153 fecal samples collected, 44 samples (28.8%) tested positive for SARS-CoV-2 compared with 126 positive results of 398 pharyngeal swabs (31.7%), which is one of the most prevalent mechanisms of testing. The rates of positivity using other specimens were 14 of 15 tests using bronchoalveolar lavage fluid (93.3%), 72 of 104 tests using sputum (69.2%), 5 of 8 tests using nasal swabs (62.5%), 6 of 13; tests using fiberoptic bronchoscope brush biopsy (46.2%), and 3 of 307 tests using blood (1.0%). Of 72 urine specimens tested, 0 were positive for SARS-CoV-2.^41^ Two stool samples (1.3%) showed live virus on electron microscopy. Xiao et al^20^ reported that, of 73 patients with SARS-CoV-2 infection, 39 patients (53.4%), including 25 men and 14 women, had results positive for SARS-CoV-2 RNA in stool specimens. Moreover, Xiao et al^20^ found that 17 patients (23.3%) had PCR of stool results positive for SARS-CoV-2 despite having respiratory samples with PCR results negative for SARS-CoV-2.

In a study by Young et al,^42^ in 4 of 8 patients had RT-PCR results positive for SARS-CoV-2 in stool, regardless of diarrhea, for 1 to 7 days. Peng et al^43^ found that anal swabs from 2 of 9 patients tested positive for SARS-CoV-2. In a single-patient case study^45^ comparing the presence of SARS-CoV-2 in throat vs rectum samples, the rectal samples were positive for SARS-CoV-2 for up to 18 days after hospitalization. A study by Ling et al^21^ included 66 patients who recovered after treatment for COVID-19, among whom 11 patients (16.7%) still had stool test results positive for viral RNA after a median duration of 11 days after symptom onset. In another study including 17 patients with confirmed SARS-CoV-2 infection with available data (representing 0-13 days after onset), stool samples from 9 patients (52.9%) were positive for SARS-CoV-2 on RT-PCR analysis for 0 to 11 days after disease onset.^7^ The viral loads in stool samples were lower than those of respiratory samples (range, 550 copies per mL to 1.21 × 10^5^ copies per mL vs 641 copies per mL to 1.34 × 10^11^ copies per mL).^7^

### Elevated Liver Enzymes

Of 23 studies included in the final analysis, 8 studies reported data on elevation of either AST or ALT levels at the time of patients’ clinical presentation. The pooled estimate of elevated AST levels was 20% (95% CI, 15.3%-25.6%) of patients, including 243 patients with elevated AST levels of 1450 patients from 8 studies (*I*^2^ = 48%; Egger test for bias, *P* = .34) (eFigure in the Supplement). The pooled prevalence of elevated ALT levels was 14.6% (95% CI, 12.8%-16.6%) of patients, including 197 patients with elevated ALT levels of 1347 patients from 6 studies (*I*^2^ = 0%; Egger test for bias, *P* = .13) (eFigure in the Supplement).

The pooled prevalence of elevated AST levels when including all studies, published and preprint, was 17.7% (95% CI, 14.1%-22%) of patients, including 272 patients with elevated AST levels of 1680 patients from 11 studies (*I*^2^ = 53%; Egger test for bias, *P* = .23). The pooled prevalence of elevated ALT was 18.5% (95% CI, 12.4%-26.5%) of patients, including 302 patients with elevated ALT levels of 1725 patients from 10 studies (*I*^2^ = 87%; Egger test for bias, *P* = .81).

## Discussion

In this systematic review and pooled analysis of the published and preprint literature of SARS-CoV-2 infection and GI symptoms, we found that approximately 10% to 12% of patients with COVID-19 experience GI symptoms, such as diarrhea (7.4%) and nausea or vomiting (4.6%). In addition, 30% to 50% of patients may have fecal swabs that test positive for the SARS-CoV-2 RNA, confirming that the virus can be detected in other sites and therefore potentially transmitted in ways other than by respiratory droplets. Particularly concerning was the presence of live virus in patients’ stool and that fecal shedding continued for days after hospitalization.

In this study, we report that the pooled prevalence of diarrhea among patients was 7.4% and of nausea or vomiting was 4.6%. Although these numbers are not as high compared with SARS infection, where the estimate of GI symptoms is approximately 20%,^46,47,48^ based on our understanding of the pathophysiological and phylogenetical characteristics of SARS-CoV-2, it might be possible that GI symptoms could be underreported in the initial studies. The reasons for this may be related to the focus on the more important and fatal respiratory symptoms being managed by treating physicians, as well as the challenges in the definition of diarrhea. Nevertheless, the presence of GI symptoms may portend a worse outcome for patients infected with SARS-CoV-2, as shown in a 2020 study by Pan et al^7^ that reported that patients without GI symptoms were more likely to recover and be discharged compared with those with GI symptoms (60% vs 34%). Liver enzyme levels outside of reference ranges have also been observed in 15% to 20% of patients. Guan et al^22^ reported significantly higher elevation in liver enzymes in patients with severe COVID-19 compared with patients with nonsevere COVID-19 (39.4% vs 18.2%). Similarly Wang et al^23^ reported higher levels in patients in intensive care units. Chen et al^24^ reported liver enzyme levels as high as 1445 U/L for AST and 7590 U/L for ALT (to convert to microkatal per liter, multiply by 0.0167).

There have been some reports^20,41^ regarding the virus being detected from other sites, and therefore potentially transmitted in ways other than respiratory droplets. In our analysis of a few studies in which SARS-CoV-2 RNA was isolated from the stool, the fecal-oral route of transmission could be an additional potential source of infection spread. Our results also suggest that testing of the virus in feces by real-time RT-PCR could be helpful in disease monitoring and surveillance. Current Centers for Disease Control and Prevention recommendations for transmission-based precautions for hospitalized patients with COVID-19 are based on having negative results on at least 2 sequential respiratory tract specimens collected at least 24 hours apart.^49^ A study by Xiao et al^20^ found that more than 20% patients with of SARS-CoV-2 infection have test results positive for viral RNA in stool even after negative conversion of viral RNA in the respiratory tract. Moreover, fecal-oral transmission of the virus could explain some of the nosocomial infections, especially those occurring in endoscopy units. Nosocomial transmission of SARS-CoV-2 is a severe problem, as 3019 health care workers in Wuhan, China, were infected as of February 12, 2020, which accounted for close to 4% of the total number of infections in Wuhan and burdened health care systems.^21,50^ To reduce health care–associated infections, clinicians should be take extra precautions when a patient reports diarrhea.

To our knowledge, this systematic review and meta-analysis offers the most up-to-date summary of the ongoing COVID-19 outbreak with burgeoning information being reported in a short time span, with focus on GI symptoms and fecal viral shedding due to COVID-19. This study provides important clinical information and insights for clinicians and epidemiologists and for future research.

### Limitations

This analysis has some limitations. First, most available studies were from China and were large case series or observational studies, which are subject to methodological biases; however, observational studies are currently the only ethical study type to measure presence of GI symptoms in patients with COVID-19. Moreover, the quality of clinical assessment, individual patient record–screening vs hospital database, was not clear in the studies included. Second, sample size calculation and prospective study of GI symptoms during the natural history of the infection and implications were not the primary aims of the studies included in our analysis, thereby limiting deeper interpretation of pooled summary estimates. Third, multiple large series of patients have not reported significant numbers of patients with GI symptoms. However, it is not entirely clear whether minor GI symptoms were underreported in patients presenting later with severe multisystem organ failure, including severe pneumonia, or that these patients actually did not have significant GI symptoms. Similarly, minor changes in liver enzyme levels during the disease trajectory may have been underreported, and studies focusing on enzyme levels could not be incorporated owing to focus of our review. This may affect the precise population mean of reported estimates, but it is difficult to arrive at such conclusions without having had true prospective, longitudinal studies of robust power with adequate follow-up. Moreover, significant heterogeneity is noted for our results. This is likely explained by clinical and methodological heterogeneity resulting from most studies being low-power case series or retrospective in nature with varied reporting of symptoms and labs. It is also due to the fact that high heterogeneity is probable representation of population distribution of the summary estimate. Although we used a random-effects model, there was still some influence on the final results. In the setting of an emerging pandemic, although most data were from 1 region of the world, early reporting of this information could still be very helpful to guide management of COVID-19.

## Conclusions

Our findings suggest that the intestinal tropism of SARS-CoV-2 is similar to previous SARS infections and that GI symptoms are a frequent manifestation of this emerging infection. It has to be noted that although GI symptoms are frequent, fever, cough, and respiratory symptoms are still the predominant type of presentation based on data from studies from China. In addition to the call for increasing awareness for this atypical presentation, these findings regarding virus shedding in feces imply that SARS-CoV-2 could be transmitted by the fecal-oral route and support consideration of stool RT-PCR testing to aid in transmission-based precautions among patients with SARS-CoV-2 infection. Finally, particularly concerning is the presence of detectable RNA in the GI tract, making the use of optimal personal protective equipment and following up-to-date national infection control guidelines highly prudent.

## Notes

Supplement. **eTable 1.** Quality Assessment of Inclusion Studies Using Methodological Index for Nonrandomized Studies **eTable 2.** Characteristic of Studies Reporting Fecal Shedding of Virus in Stool **eFigure.** Forest Plot of Included Studies Showing Pooled Estimate of Elevated Aspartate Aminotransferase and Alanine Aminotransferase **eReferences**

## References

[1] Fan Z, Chen L, Li J, Clinical features of COVID-19-related liver damage. Clin Gastroenterol Hepatol. 2020;3565(20):30482-1. doi:10.1016/j.cgh.2020.04.002 32283325 PMC7194865

[2] Lu R, Zhao X, Li J, Genomic characterisation and epidemiology of 2019 novel coronavirus: implications for virus origins and receptor binding. Lancet. 2020;395(10224):565-574. doi:10.1016/S0140-6736(20)30251-8 32007145 PMC7159086

[3] Wu Z, McGoogan JM Characteristics of and important lessons from the coronavirus disease 2019 (COVID-19) outbreak in China: summary of a report of 72 314 cases from the Chinese Center for Disease Control and Prevention. JAMA. Published online February 24, 2020. doi:10.1001/jama.2020.2648 32091533

[4] Wang Z, Chen X, Lu Y, Chen F, Zhang W Clinical characteristics and therapeutic procedure for four cases with 2019 novel coronavirus pneumonia receiving combined Chinese and Western medicine treatment. Biosci Trends. 2020;14(1):64-68. doi:10.5582/bst.2020.01030 32037389

[5] Perlman S, Netland J Coronaviruses post-SARS: update on replication and pathogenesis. Nat Rev Microbiol. 2009;7(6):439-450. doi:10.1038/nrmicro2147 19430490 PMC2830095

[6] Zhou P, Yang XL, Wang XG, A pneumonia outbreak associated with a new coronavirus of probable bat origin. Nature. 2020;579(7798):270-273. doi:10.1038/s41586-020-2012-7 32015507 PMC7095418

[7] Pan Y, Zhang D, Yang P, Poon LLM, Wang Q Viral load of SARS-CoV-2 in clinical samples. Lancet Infect Dis. 2020;20(4):411-412. doi:10.1016/S1473-3099(20)30113-4 32105638 PMC7128099

[8] Zhang H, Kang Z, Gong H, The digestive system is a potential route of 2019-nCov infection: a bioinformatics analysis based on single-cell transcriptomes. Preprint. Posted online January 31, 2020. bioRxiv 2020.01.30.927806. doi:10.1101/2020.01.30.927806

[9] Zhou J, Li C, Zhao G, Human intestinal tract serves as an alternative infection route for Middle East respiratory syndrome coronavirus. Sci Adv. 2017;3(11):eaao4966. doi:10.1126/sciadv.aao4966 29152574 PMC5687858

[10] Zhang J, Wang S, Xue Y Fecal specimen diagnosis 2019 novel coronavirus-infected pneumonia. J Med Virol. Published online March 3, 2020. doi:10.1002/jmv.25742 32124995 PMC7228355

[11] Liang W, Feng Z, Rao S, Diarrhea may be underestimated: a missing link in 2019 novel coronavirus. Preprint. Posted online February 17, 2020. medRxiv 2020.02.03.20020289. doi:10.1101/2020.02.03.20020289 32102928

[12] Holshue ML, DeBolt C, Lindquist S, ; Washington State 2019-nCoV Case Investigation Team First case of 2019 novel coronavirus in the United States. N Engl J Med. 2020;382(10):929-936. doi:10.1056/NEJMoa2001191 32004427 PMC7092802

[13] Moher D, Shamseer L, Clarke M, ; PRISMA-P Group Preferred reporting items for systematic review and meta-analysis protocols (PRISMA-P) 2015 statement. Syst Rev. 2015;4:1. doi:10.1186/2046-4053-4-1 25554246 PMC4320440

[14] Yang W, Cao Q, Qin L, Clinical characteristics and imaging manifestations of the 2019 novel coronavirus disease (COVID-19):A multi-center study in Wenzhou city, Zhejiang, China. J Infect. 2020;80(4):388-393. doi:10.1016/j.jinf.2020.02.016 32112884 PMC7102539

[15] Allen Institute for AI CORD-19: COVID-19 Open Research Dataset. Accessed May 15, 2020. https://www.semanticscholar.org/cord19

[16] Slim K, Nini E, Forestier D, Kwiatkowski F, Panis Y, Chipponi J Methodological index for non-randomized studies (minors): development and validation of a new instrument. ANZ J Surg. 2003;73(9):712-716. doi:10.1046/j.1445-2197.2003.02748.x 12956787

[17] Xu XW, Wu XX, Jiang XG, Clinical findings in a group of patients infected with the 2019 novel coronavirus (SARS-Cov-2) outside of Wuhan, China: retrospective case series. BMJ. 2020;368:m606. doi:10.1136/bmj.m606 32075786 PMC7224340

[18] Chen H, Guo J, Wang C, Clinical characteristics and intrauterine vertical transmission potential of COVID-19 infection in nine pregnant women: a retrospective review of medical records. Lancet. 2020;395(10226):809-815. doi:10.1016/S0140-6736(20)30360-3 32151335 PMC7159281

[19] Wang D, Ju XL, Xie F, Clinical analysis of 31 cases of 2019 novel coronavirus infection in children from six provinces (autonomous region) of northern China [in Chinese]. Zhonghua Er Ke Za Zhi. 2020;58(4):269-274. doi:10.3760/cma.j.cn112140-20200225-00138 32118389

[20] Xiao F, Tang M, Zheng X, Liu Y, Li X, Shan H Evidence for gastrointestinal infection of SARS-CoV-2. Gastroenterology. 2020;158(6):1831-1833.e3. doi:10.1053/j.gastro.2020.02.055 32142773 PMC7130181

[21] Ling Y, Xu SB, Lin YX, Persistence and clearance of viral RNA in 2019 novel coronavirus disease rehabilitation patients. Chin Med J (Engl). 2020;133(9):1039-1043. doi:10.1097/CM9.0000000000000774 32118639 PMC7147278

[22] Guan WJ, Ni ZY, Hu Y, ; China Medical Treatment Expert Group for Covid-19 Clinical characteristics of coronavirus disease 2019 in China. N Engl J Med. 2020;382(18):1708-1720. doi:10.1056/NEJMoa2002032 32109013 PMC7092819

[23] Wang D, Hu B, Hu C, Clinical Characteristics of 138 Hospitalized Patients With 2019 Novel Coronavirus-Infected Pneumonia in Wuhan, China. JAMA. 2020;323(11):1061‐1069. doi:10.1001/jama.2020.1585 32031570 PMC7042881

[24] Chen N, Zhou M, Dong X, Epidemiological and clinical characteristics of 99 cases of 2019 novel coronavirus pneumonia in Wuhan, China: a descriptive study. Lancet. 2020;395(10223):507-513. doi:10.1016/S0140-6736(20)30211-7 32007143 PMC7135076

[25] Liu K, Fang YY, Deng Y, Clinical characteristics of novel coronavirus cases in tertiary hospitals in Hubei Province. Chin Med J (Engl). 2020;133(9):1025-1031. doi:10.1097/CM9.0000000000000744 32044814 PMC7147277

[26] Liu M, He P, Liu HG, Clinical characteristics of 30 medical workers infected with new coronavirus pneumonia [in Chinese]. Zhonghua Jie He He Hu Xi Za Zhi. 2020;43(3):209-214. doi:10.3760/cma.j.issn.1001-0939.2020.03.014 32164090

[27] Huang C, Wang Y, Li X, Clinical features of patients infected with 2019 novel coronavirus in Wuhan, China. Lancet. 2020;395(10223):497-506. doi:10.1016/S0140-6736(20)30183-5 31986264 PMC7159299

[28] Zhang MQ, Wang XH, Chen YL, Clinical features of 2019 novel coronavirus pneumonia in the early stage from a fever clinic in Beijing [in Chinese]. Zhonghua Jie He Hu Xi Za Zhi. 2020;43(3):215-218. doi:10.3760/cma.j.issn.1001-0939.2020.03.015 32164091

[29] Chen L, Liu HG, Liu W, Analysis of clinical features of 29 patients with 2019 novel coronavirus pneumonia [in Chinese]. Zhonghua Jie He He Hu Xi Za Zhi. 2020;43(3):203-208. 32164089 10.3760/cma.j.issn.1001-0939.2020.03.013

[30] Chang D, Lin M, Wei L, Epidemiologic and clinical characteristics of novel coronavirus infections involving 13 patients outside Wuhan, China. JAMA. 2020;323(11):1092-1093. doi:10.1001/jama.2020.1623 32031568 PMC7042871

[31] Pan L, Mu M, Yang P, Clinical characteristics of COVID-19 patients with digestive symptoms in Hubei, China: a descriptive, cross-sectional, multicenter study. Am J Gastroenterol. 2020;115(5):766-773. doi:10.14309/ajg.0000000000000620 32287140 PMC7172492

[32] Luo S, Zhang X, Xu H Don’t overlook digestive symptoms in patients with 2019 novel coronavirus disease (COVID-19). Clin Gastroenterol Hepatol. 2020;S1542-3565(20)30401-30408. doi:10.1016/j.cgh.2020.03.043 32205220 PMC7154217

[33] Zhou Z, Zhao N, Shu Y, Han S, Chen B, Shu X Effect of gastrointestinal symptoms on patients infected with coronavirus disease 2019. Gastroenterology. 2020;S0016-5085(20)30362-30370. doi:10.1053/j.gastro.2020.03.020 32199880 PMC7270807

[34] Jin X, Lian JS, Hu JH, Epidemiological, clinical and virological characteristics of 74 cases of coronavirus-infected disease 2019 (COVID-19) with gastrointestinal symptoms. Gut. 2020;69(6):1002-1009. doi:10.1136/gutjnl-2020-320926 32213556 PMC7133387

[35] Zhang C, Gu J, Chen Q, Clinical characteristics of 34 children with coronavirus disease-2019 in the west of China: a multiple-center case series. Preprint. Posted online March 16, 2020. medRxiv 2020.03.12.20034686. doi:10.1101/2020.03.12.20034686

[36] Xu Y, Li X, Zhu B, Characteristics of pediatric SARS-CoV-2 infection and potential evidence for persistent fecal viral shedding. Nat Med. 2020;26(4):502-505. doi:10.1038/s41591-020-0817-4 32284613 PMC7095102

[37] Qian G-Q, Yang NB, Ding F, Epidemiologic and clinical characteristics of 91 hospitalized patients with COVID-19 in Zhejiang, China: a retrospective, multi-centre case series. QJM. Published online March 17, 2020. doi:10.1093/qjmed/hcaa089 32181807 PMC7184349

[38] Zhao Z, Xie J, Yin M, Clinical and laboratory profiles of 75 hospitalized patients with novel coronavirus disease 2019 in Hefei, China. Preprint. Posted online March 6, 2020. medRxiv 2020.03.01.20029785. doi:10.1101/2020.03.01.20029785

[39] Han C, Duan C, Zhang S, Digestive symptoms in COVID-19 patients with mild disease severity: clinical presentation, stool viral RNA testing, and outcomes. Am J Gastroenterol. 2020. doi:10.14309/ajg.0000000000000664 32301761 PMC7172493

[40] Liu J, Ouyang L, Guo P, Epidemiological, clinical characteristics and outcome of medical staff infected with COVID-19 in Wuhan, China: a retrospective case series analysis. Preprint. Posted online March 13, 2020. medRxiv 2020.03.09.20033118. doi:10.1101/2020.03.09.20033118

[41] Wang W, Xu Y, Gao R, Detection of SARS-CoV-2 in different types of clinical specimens. JAMA. 2020;323(18):1843-1844. doi:10.1001/jama.2020.3786 32159775 PMC7066521

[42] Young BE, Ong SWX, Kalimuddin S, ; Singapore 2019 Novel Coronavirus Outbreak Research Team Epidemiologic features and clinical course of patients infected with SARS-CoV-2 in Singapore. JAMA. 2020;323(15):1488-1494. doi:10.1001/jama.2020.3204 32125362 PMC7054855

[43] Peng L, Liu J, Xu W, Luo Q, Deng K, Lin B, Gao Z 2019 Novel coronavirus can be detected in urine, blood, anal swabs and oropharyngeal swabs samples. Preprint. Posted online February 25, 2020. medRxiv 2020.02.21.20026179. doi:10.1101/2020.02.21.20026179

[44] Wu Y, Guo C, Tang L, Prolonged presence of SARS-CoV-2 viral RNA in faecal samples. Lancet Gastroenterol Hepatol. 2020;5(5):434-435. doi:10.1016/S2468-1253(20)30083-2 32199469 PMC7158584

[45] Tan LV, Ngoc NM, That BTT, Duration of viral detection in throat and rectum of a patient with COVID-19. Preprint. Posted online March 16, 2020. medRxiv 2020.03.07.20032052. doi:10.1101/2020.03.07.20032052

[46] Chiu YC, Wu KL, Chou YP, Diarrhea in medical care workers with severe acute respiratory syndrome. J Clin Gastroenterol. 2004;38(10):880-882. doi:10.1097/00004836-200411000-00009 15492605

[47] Leung WK, To KF, Chan PK, Enteric involvement of severe acute respiratory syndrome-associated coronavirus infection. Gastroenterology. 2003;125(4):1011-1017. doi:10.1016/j.gastro.2003.08.001 14517783 PMC7126982

[48] Shi X, Gong E, Gao D, Severe acute respiratory syndrome associated coronavirus is detected in intestinal tissues of fatal cases. Am J Gastroenterol. 2005;100(1):169-176. doi:10.1111/j.1572-0241.2005.40377.x 15654797

[49] Centers for Disease Control and Prevention Discontinuation of transmission-based precautions and disposition of patients with COVID-19 in healthcare settings (interim guidance). Accessed April 24, 2020. https://www.cdc.gov/coronavirus/2019-ncov/hcp/disposition-hospitalized-patients.html

[50] Wang Y, Wang Y, Chen Y, Qin Q Unique epidemiological and clinical features of the emerging 2019 novel coronavirus pneumonia (COVID-19) implicate special control measures. J Med Virol. Published online March 5, 2020. doi:10.1002/jmv.25748 32134116 PMC7228347

