# Supplementary material for: Prevalence of Gastrointestinal Symptoms and Fecal Viral Shedding in Patients With Coronavirus Disease 2019: A Systematic Review and Meta-analysis
Source: JAMA Netw Open. 2020 Jun 11;3(6):e2011335. doi: 10.1001/jamanetworkopen.2020.11335 (PMC7290409; doi:10.1001/jamanetworkopen.2020.11335)
Supplement: Supplement. — eTable 1. Quality Assessment of Inclusion Studies Using Methodological Index for Nonrandomized Studies eTable 2. Characteristic of Studies Reporting Fecal Shedding of Virus in Stool eFigure. Forest Plot of Included Studies Showing Pooled Estimate of Elevated Aspartate Aminotransferase and Alanine Aminotransferase eReferences [file jamanetwopen-3-e2011335-s001.pdf]

Supplementary Online Content

Parasa S, Desai M, Thoguluva Chandrasekar V, et al. Prevalence of gastrointestinal symptoms and fecal viral shedding in patients with coronavirus disease 2019: a systematic review and meta-analysis. *JAMA Netw Open*. 2020;3(6):e2011335.
doi:10.1001/jamanetworkopen.2020.11335

eTable 1. Quality Assessment of Inclusion Studies Using Methodological Index for Nonrandomized Studies

eTable 2. Characteristic of Studies Reporting Fecal Shedding of Virus in Stool

eFigure. Forest Plot of Included Studies Showing Pooled Estimate of Elevated Aspartate Aminotransferase and Alanine Aminotransferase

eReferences

This supplementary material has been provided by the authors to give readers additional information about their work.

© 2020 Parasa S et al. *JAMA Network Open.*

eTable 1. Quality Assessment of Inclusion Studies Using Methodological Index for Nonrandomized Studies

Study	Stated aim of the study	Inclusion of consecutive patients	Prospective collection of data	Endpoint appropriate to study aim	Unbiased evaluation of endpoints	Follow up period appropriate to the major end point	Loss to follow up not exceeding 5%	Prospective calculation of study size	Total
Chen et al[18])	2	1	0	2	1	2	0	0	8
Zhang et al[10]	1	1	0	1	1	2	0	0	6
Wang et al[19]	2	1	0	1	1	2	0	0	7
Xiao Fei et al[35]	2	1	2	2	1	2	0	0	10
Ling et al[27]	1	2	0	2	1	2	0	0	8
Liu et al[37]	2	2	0	2	1	2	0	0	9
Yang et al[14]	2	2	0	2	1	2	0	0	9
Xu et al[17]	2	1	0	2	1	2	0	0	8
Liu et al[38]	2	1	0	2	1	2	0	0	6
Guan WJ et al[31]	1	2	0	2	1	2	2	0	10
Huang C et al[39]	2	2	2	2	1	2	2	0	13
Chen N et al[50]	2	2	0	2	1	2	0	0	9
Wang D et al[32]	2	2	0	2	1	2	0	0	9
Zhang MQ et al[40]	2	1	0	2	1	2	0	0	8
Chen L et al[50]	2	1	0	1	1	2	0	0	7
Wang Z et al[5]	1	0	0	2	1	2	0	0	6
Chang et al[42]	1	1	2	2	1	2	0	0	9
AJG Pan et al-preprint[43]	2	2	0	2	1	2	0	0	9
Xu et al[17]	1	1	1	1	0	1	0	0	5
Wu et al[4]	1	1	1	2	0	2	0	0	7
Jin et al[47]	2	2	0	2	0	2	0	0	8

© 2020 Parasa S et al. *JAMA Network Open.*

**Luo et al[44]**	1	2	0	1	0	1	0	0	5
**Zhou et al[45]**	1	2	1	1	0	2	0	0	7
**Han et al[49]**	2	2	0	2	0	2	0	0	8

© 2020 Parasa S et al. *JAMA Network Open.*

eTable 2. Characteristic of Studies Reporting Fecal Shedding of Virus in Stool

Author	Publication date	Country/Hospital	Age (median, IQR)	Gender-female	Time period	Number of patients	Diarrhea (at presentation)	Fecal shedding in confirmed cases with respiratory symptoms
Xiao Fei et al, 2020[23]	March 3, 2020	Sun Yat-sen University, China	10 months to 78 year old	14 (19%)	February 1 to 14, 2020	73	26 (35.6%); 10 (13.6%) GI bleeding	39/73 (53.42%)
Zhang et al, 2020[10]	March 3, 2020	Jinhua Hospital of Zhejiang University	41 years (18–87 years)	7 (50%)	27 January 2020 to 10 February 2020	14	0	5/14 (35.7%)
Young et al, 2020[24]	Mar 3, 2020	4 hospitals in Singapore	47 years	9 (50%)	January 23, 2020 to February 3, 2020	18	Not clearly reported	4/8 (50%)
Peng et al, 2020[25]	February 25, 2020	Affiliated Hospital of Sun Yat-sen University, China	27-62 years	5 (55.56 %)	Not reported	9	1(11%)	2/9 (22%)
Wu et al, 2020[51]	March 19, 2020	Fifth Affiliated Hospital at Sun-Yat Sen University	Not reported	Not reported	January 16 – March 15, 2020	74	Not reported	41 (55.4%)
Ling et al, 2020[27]	February 28, 2020	Shanghai Public Clinic health center	44 (34-62 years)	28 (42.4%)	January 20 – February 20, 2020	66	Not reported	11 (16.7%)
Xu et al, 2020[17]	March 13, 2020	Guangzhou Women and Children's medical center	2 months – 15 years	4 (40%)	January 22 – February 20, 2020	10	2 (20%)	8 (80%)
Wang et al, 2020[22]	March 11, 2020	3 hospitals in the Hubei and Shandong provinces and Beijing, China	44 (5-67) years	66 (32%)	January 1, 2020 to February 17, 2020	205	Not reported	44/153 (29%)

© 2020 Parasa S et al. *JAMA Network Open.*

eFigure. Forest Plot of Included Studies Showing Pooled Estimate of Elevated Aspartate Aminotransferase and Alanine Aminotransferase

Supplementary Figure 1: Forest plot of inclusion studies showing pooled estimate of elevated AST and ALT among published studies (A) and all studies (B)

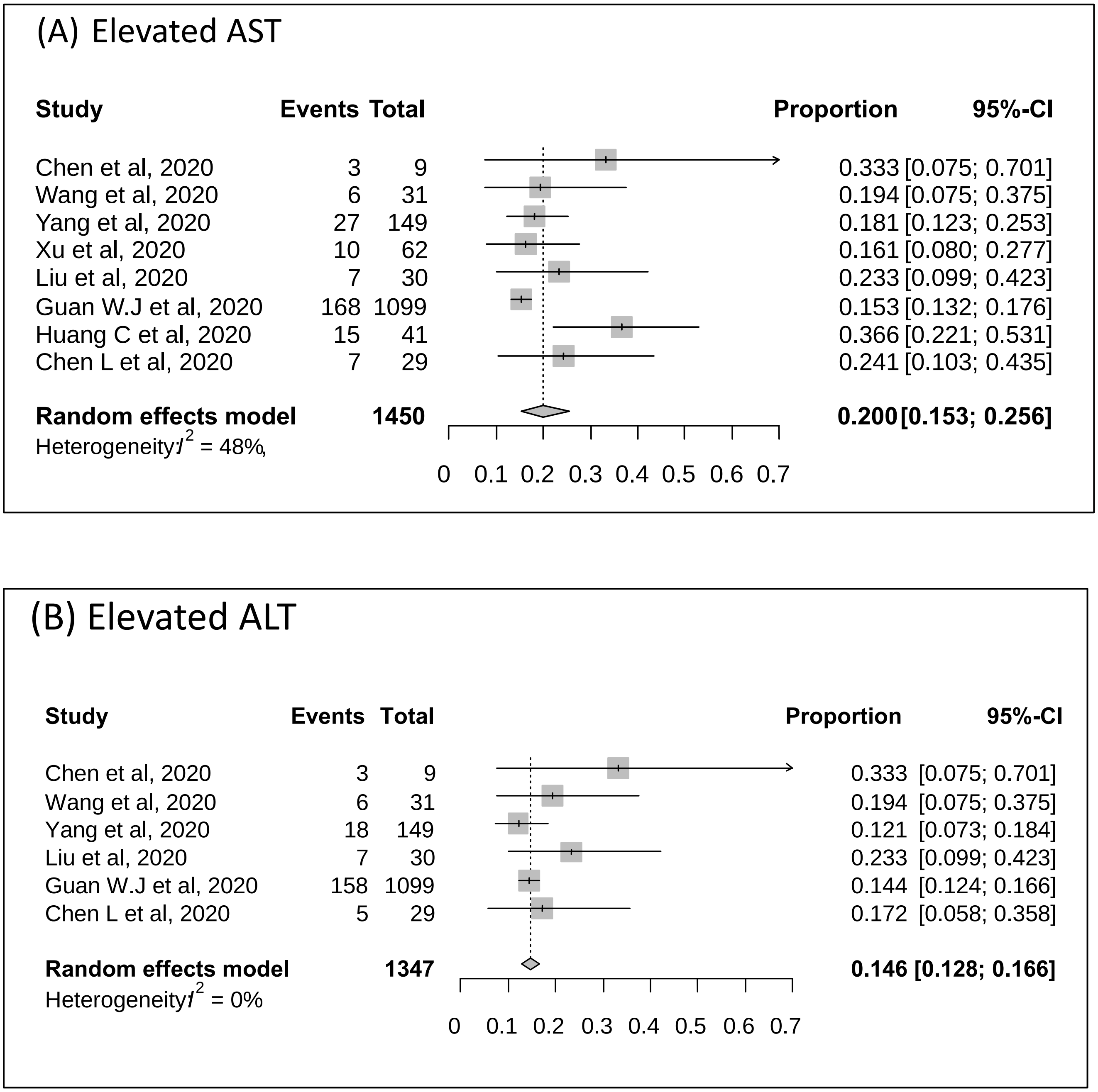

© 2020 Parasa S et al. *JAMA Network Open.*

eReferences

1. Fan, Z., et al., *Clinical Features of COVID-19 Related Liver Damage.* medRxiv, 2020: p. 2020.02.26.20026971.
2. Zhou, P., et al., *A pneumonia outbreak associated with a new coronavirus of probable bat origin.* Nature, 2020. **579**(7798): p. 270-273.
3. Lu, R., et al., *Genomic characterisation and epidemiology of 2019 novel coronavirus: implications for virus origins and receptor binding.* Lancet, 2020. **395**(10224): p. 565-574.
4. Wu, Z. and J.M. McGoogan, *Characteristics of and Important Lessons From the Coronavirus Disease 2019 (COVID-19) Outbreak in China: Summary of a Report of 72314 Cases From the Chinese Center for Disease Control and Prevention.* Jama, 2020.
5. Wang, Z., et al., *Clinical characteristics and therapeutic procedure for four cases with 2019 novel coronavirus pneumonia receiving combined Chinese and Western medicine treatment.* Biosci Trends, 2020. **14**(1): p. 64-68.
6. Perlman, S. and J. Netland, *Coronaviruses post-SARS: update on replication and pathogenesis.* Nat Rev Microbiol, 2009. **7**(6): p. 439-50.
7. Pan, Y., et al., *Viral load of SARS-CoV-2 in clinical samples.* Lancet Infect Dis, 2020.
8. Zhang, H., et al., *The digestive system is a potential route of 2019-nCov infection: a bioinformatics analysis based on single-cell transcriptomes.* bioRxiv, 2020: p. 2020.01.30.927806.
9. Zhou, J., et al., *Human intestinal tract serves as an alternative infection route for Middle East respiratory syndrome coronavirus.* Science Advances, 2017. **3**(11): p. eaao4966.
10. Zhang, J., S. Wang, and Y. Xue, *Fecal specimen diagnosis 2019 novel coronavirus-infected pneumonia.* J Med Virol, 2020.
11. Liang, W., et al., *Diarrhea may be underestimated: a missing link in 2019 novel coronavirus.* medRxiv, 2020: p. 2020.02.03.20020289.
12. Holshue, M.L., et al., *First Case of 2019 Novel Coronavirus in the United States.* N Engl J Med, 2020. **382**(10): p. 929-936.
13. Moher, D., et al., *Preferred reporting items for systematic review and meta-analysis protocols (PRISMA-P) 2015 statement.* Syst Rev, 2015. **4**: p. 1.
14. Yang, W., et al., *Clinical characteristics and imaging manifestations of the 2019 novel coronavirus disease (COVID-19):A multi-center study in Wenzhou city, Zhejiang, China.* J Infect, 2020.
15. *COVID-19 Open Research Dataset (CORD-19). 2020. Version 2020-03-13.* https://pages.semanticscholar.org/coronavirus-research.
16. Slim, K., et al., *Methodological index for non-randomized studies (minors): development and validation of a new instrument.* ANZ J Surg, 2003. **73**(9): p. 712-6.
17. Xu, X.W., et al., *Clinical findings in a group of patients infected with the 2019 novel coronavirus (SARS-Cov-2) outside of Wuhan, China: retrospective case series.* Bmj, 2020. **368**: p. m606.
18. Chen, H., et al., *Clinical characteristics and intrauterine vertical transmission potential of COVID-19 infection in nine pregnant women: a retrospective review of medical records.* Lancet, 2020. **395**(10226): p. 809-815.
19. Wang, D., et al., *[Clinical analysis of 31 cases of 2019 novel coronavirus infection in children from six provinces (autonomous region) of northern China].* Zhonghua Er Ke Za Zhi, 2020. **58**(4): p. E011.
20. Zhang, C., et al., *Clinical Characteristics of 34 Children with Coronavirus Disease-2019 in the West of China: a Multiple-center Case Series.* medRxiv, 2020: p. 2020.03.12.20034686.
21. Xu, Y., et al., *Characteristics of pediatric SARS-CoV-2 infection and potential evidence for persistent fecal viral shedding.* Nature Medicine, 2020.

© 2020 Parasa S et al. *JAMA Network Open.*

22. Wang, W., et al., *Detection of SARS-CoV-2 in Different Types of Clinical Specimens.* JAMA, 2020.
23. Xiao, F., et al., *Evidence for gastrointestinal infection of SARS-CoV-2.* Gastroenterology, 2020.
24. Young, B.E., et al., *Epidemiologic Features and Clinical Course of Patients Infected With SARS-CoV-2 in Singapore.* Jama, 2020.
25. Peng, L., et al., *2019 Novel Coronavirus can be detected in urine, blood, anal swabs and oropharyngeal swabs samples.* medRxiv, 2020: p. 2020.02.21.20026179.
26. Tan, L.V., et al., *Duration of viral detection in throat and rectum of a patient with COVID-19.* medRxiv, 2020: p. 2020.03.07.20032052.
27. Ling, Y., et al., *Persistence and clearance of viral RNA in 2019 novel coronavirus disease rehabilitation patients.* Chin Med J (Engl), 2020.
28. Chiu, Y.C., et al., *Diarrhea in medical care workers with severe acute respiratory syndrome.* J Clin Gastroenterol, 2004. **38**(10): p. 880-2.
29. Leung, W.K., et al., *Enteric involvement of severe acute respiratory syndrome-associated coronavirus infection.* Gastroenterology, 2003. **125**(4): p. 1011-7.
30. Shi, X., et al., *Severe acute respiratory syndrome associated coronavirus is detected in intestinal tissues of fatal cases.* Am J Gastroenterol, 2005. **100**(1): p. 169-76.
31. Guan, W.J., et al., *Clinical Characteristics of Coronavirus Disease 2019 in China.* N Engl J Med, 2020.
32. Wang, D., et al., *Clinical Characteristics of 138 Hospitalized Patients With 2019 Novel Coronavirus-Infected Pneumonia in Wuhan, China.* Jama, 2020.
33. Chen, N., et al., *Epidemiological and clinical characteristics of 99 cases of 2019 novel coronavirus pneumonia in Wuhan, China: a descriptive study.* Lancet, 2020. **395**(10223): p. 507-513.
34. *Discontinuation of Transmission-Based Precautions and Disposition of Patients with COVID-19 in Healthcare Settings (Interim Guidance).* [cited 2020 April 24]; Available from: https://www.cdc.gov/coronavirus/2019-ncov/hcp/disposition-hospitalized-patients.html.
35. Xiao, F., et al., *Evidence for gastrointestinal infection of SARS-CoV-2.* medRxiv, 2020: p. 2020.02.17.20023721.
36. Wang, Y., et al., *Unique epidemiological and clinical features of the emerging 2019 novel coronavirus pneumonia (COVID-19) implicate special control measures.* J Med Virol, 2020.
37. Liu, K., et al., *Clinical characteristics of novel coronavirus cases in tertiary hospitals in Hubei Province.* Chin Med J (Engl), 2020.
38. Liu, M., et al., *[Clinical characteristics of 30 medical workers infected with new coronavirus pneumonia].* Zhonghua Jie He He Hu Xi Za Zhi, 2020. **43**(3): p. 209-214.
39. Huang, C., et al., *Clinical features of patients infected with 2019 novel coronavirus in Wuhan, China.* Lancet, 2020. **395**(10223): p. 497-506.
40. Zhang, M.Q., et al., *[Clinical features of 2019 novel coronavirus pneumonia in the early stage from a fever clinic in Beijing].* Zhonghua Jie He He Hu Xi Za Zhi, 2020. **43**(3): p. 215-218.
41. Chen, L., et al., *[Analysis of clinical features of 29 patients with 2019 novel coronavirus pneumonia].* Zhonghua Jie He He Hu Xi Za Zhi, 2020. **43**(3): p. 203-208.
42. Chang, et al., *Epidemiologic and Clinical Characteristics of Novel Coronavirus Infections Involving 13 Patients Outside Wuhan, China.* Jama, 2020.
43. Lei Pan, M.M., MD, Hong Gang Ren, Pengcheng Yang, Yu Sun, Rungsheng Wang, Junhong Yan, Pibao Li,, Baoguang Hu, Chaohu,Yuan Jin, Xun Niu, Rongyu Ping,Yingzhen Du,Tianzhi Li, Chengxia Liu, Guogang Xu,Qinyong Hu, and L. Tu, *Clinical characteristics of COVID-19 patients with digestive yymptoms in Hubei, China: a descriptive, cross-sectional, multicenter study.* The American Journal of Gastroenterology, 2020.
44. Luo S, Z.X., Xu H, *Don't overlook digestive symptoms in patients with*

© 2020 Parasa S et al. *JAMA Network Open.*

2019 novel coronavirus disease (COVID-19). Clinical Gastroenterology and Hepatology (2020), doi: https://doi.org/10.1016/j.cgh.2020.03.043., 2020.
45. Zhou Z, Z.N., Shu Y, Han S, Chen B, Shu X, *Effect of gastrointestinal symptoms on patients infected with COVID-19.* Gastroenterology (2020), doi: https://doi.org/10.1053/j.gastro.2020.03.020, 2020.
46. Jin, X., et al., *Epidemiological, clinical and virological characteristics of 74 cases of coronavirus-infected disease 2019 (COVID-19) with gastrointestinal symptoms.* Gut, 2020: p. gutjnl-2020-320926.
47. Qian, G.-Q., et al., *Epidemiologic and Clinical Characteristics of 91 Hospitalized Patients with COVID-19 in Zhejiang, China: A retrospective, multi-centre case series.* medRxiv, 2020: p. 2020.02.23.20026856.
48. Zhao, Z., et al., *Clinical and Laboratory Profiles of 75 Hospitalized Patients with Novel Coronavirus Disease 2019 in Hefei, China.* medRxiv, 2020: p. 2020.03.01.20029785.
49. Chaoqun Han, C.D., Shengyan Zhang, Brennan Spiegel, Huiying Shi,Weijun Wang, Lei Zhang, Rong Lin, Jun Liu, Zhen Ding, Xiaohua Hou, *Digestive Symptoms in COVID-19 Patients with Mild Disease Severity: Clinical Presentation, Stool Viral RNA Testing, and Outcomes.* American Journal of Gastroenterology - Preprint.
50. Liu, J., et al., *Epidemiological, Clinical Characteristics and Outcome of Medical Staff Infected with COVID-19 in Wuhan, China: A Retrospective Case Series Analysis.* medRxiv, 2020: p. 2020.03.09.20033118.
51. Wu, Y., et al., *Prolonged presence of SARS-CoV-2 viral RNA in faecal samples.* Lancet Gastroenterol Hepatol, 2020. **5**(5): p. 434-435.

© 2020 Parasa S et al. *JAMA Network Open.*
